# The Use of *Hibiscus esculentus* (Okra) Gum in Sustaining the Release of Propranolol Hydrochloride in a Solid Oral Dosage Form

**DOI:** 10.1155/2014/735891

**Published:** 2014-02-11

**Authors:** Nurul Dhania Zaharuddin, Mohamed Ibrahim Noordin, Ali Kadivar

**Affiliations:** Department of Pharmacy, Medical Faculty, University of Malaya, 50603 Kuala Lumpur, Malaysia

## Abstract

The effectiveness of Okra gum in sustaining the release of propranolol hydrochloride in a tablet was studied. Okra gum was extracted from the pods of *Hibiscus esculentus* using acetone as a drying agent. Dried Okra gum was made into powder form and its physical and chemical characteristics such as solubility, pH, moisture content, viscosity, morphology study using SEM, infrared study using FTIR, crystallinity study using XRD, and thermal study using DSC and TGA were carried out. The powder was used in the preparation of tablet using granulation and compression methods. Propranolol hydrochloride was used as a model drug and the activity of Okra gum as a binder was compared by preparing tablets using a synthetic and a semisynthetic binder which are hydroxylmethylpropyl cellulose (HPMC) and sodium alginate, respectively. Evaluation of drug release kinetics that was attained from dissolution studies showed that Okra gum retarded the release up to 24 hours and exhibited the longest release as compared to HPMC and sodium alginate. The tensile and crushing strength of tablets was also evaluated by conducting hardness and friability tests. Okra gum was observed to produce tablets with the highest hardness value and lowest friability. Hence, Okra gum was testified as an effective adjuvant to produce favourable sustained release tablets with strong tensile and crushing strength.

## 1. Introduction

Natural polymers are useful polysaccharides obtained from plants that could be specifically applied in various pharmaceutical products. In the current era, they have been extracted widely from rain fed and irrigated crop production that could yield polysaccharides which can be applied in not only the pharmaceutical field, but also food and other processing industries. Polysaccharides are considered to be feasible raw materials as they are from renewable sources, stable, and widely available in many countries [1].

Okra gum from the pods of *Hibiscus esculentus* is one of the advantageous polysaccharides that is currently being studied in the pharmaceutical industry as a hydrophilic polymer in pharmaceutical dosage forms. Okra plant grows very fast, is grown in all soil types, and is among the most heat and drought-tolerant vegetables [2]. It has been investigated as a binding agent for tablets and has also been shown to produce tablets with good hardness, friability, and drug release profiles [3]. It has advantage over most commercial synthetic polymers as it is safe, chemically inert, nonirritant, biodegradable, biocompatible, and eco-friendly. Since it is widely harvested and does not require toxicology studies, it is therefore considered to be economical [4].

**Figure 1:**
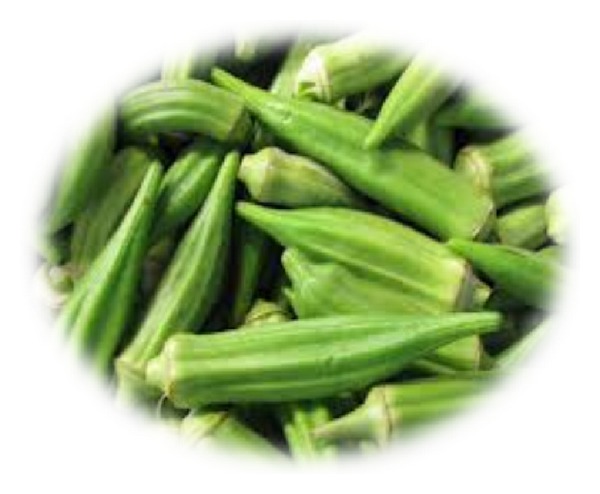
Okra fruit.

**Figure 2:**
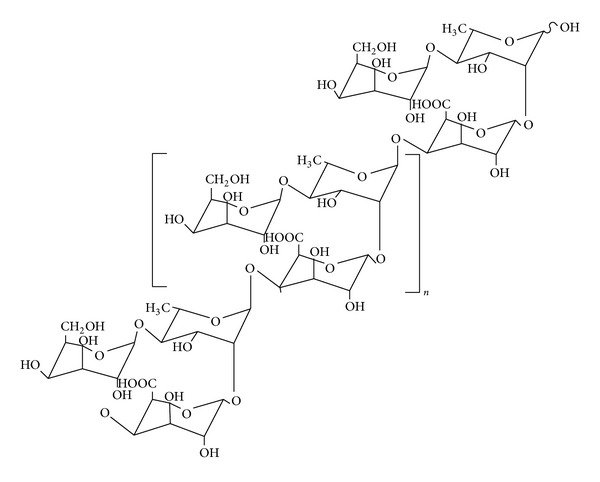
Chemical structure of polysaccharide of Okra mucilage.

Okra gum contains random coil polysaccharides consisting of galactose, rhamnose, and galacturonic acid (Figure 2). The repeating units of the gum were found to be (1-2)-rhamnose and (1-4)-galacturonic acid residues with disaccharide side chains and a degree of acetylation (DA = 58) [5]. When extracted in water, these polysaccharides can produce highly viscous solution with a slimy appearance. Therefore, the highly viscous property of Okra gum may be useful as a retarding polymer in the formulation of sustained release tablets (Figure 1).

Propranolol hydrochloride was chosen as a model drug in this study. It is an antihypertensive, antianginal, and antiarrhythmic, agent and is also used for the treatment of migraine. Propranolol HCl has a short half-life (3-4 hours) and is an acid-soluble basic drug [6]. Due to these characteristics, it was selected as a model drug for sustained release tablets for gastric retention.

In this study, the binding and retarding behaviour of Okra gum which plays an important role in the formulation of sustained release drug delivery system was investigated.

## 2. Materials and Methods

### 2.1. Materials and Chemicals

Okra fruits were obtained from the local market. Propranolol hydrochloride was a gift sample from Prima Interchem Malaysia. Hydroxylmethylpropyl cellulose (E15 LV Premium), sodium alginate (5–40 cps), and lactose were obtained from R&M Marketing, Essex, UK. Calcium chloride anhydrous, acetone, 2-propanol, and hydrochloric acid 37% were purchased from Friendemann Schmidt Chemical. Magnesium stearate and Polyethylene glycol 4000 were obtained from Merck-Schuchardt (Figure 5). All chemicals are of analytical grade.

### 2.2. Extraction of Okra Gum

The extraction of Okra gum method was modified based on the procedure by Tavakoli et al. [7].

1 kg of unripe and tender Okra fruits (pods) was obtained from the local market. The seeds were removed as they do not contain any mucilage. The fruits were washed and sliced thinly with a knife. The sliced mass was soaked in distilled water overnight to extract out the mucilage. After soaking, a white muslin cloth was used to filter out the viscous gum extract (mucilage). Acetone was added to precipitate the gum at a ratio of 3 parts of acetone to 1 part of the gum extract. Then, the precipitated gum was dried in a desiccator containing anhydrous calcium chloride for approximately 2 weeks. Size reduction and screening of the dried gum were carried out using a stainless steel grinder and no. 30 stainless steel mesh sieve. Airtight powder bottles were used to store the undersized fractions. Subsequently, physicochemical characterization of the Okra gum powder was conducted.

### 2.3. Characterization of Extracted Okra Gum

Based on previous studies, experiments were modified from the literature and conducted in accordance with British Pharmacopeia 2007.

#### 2.3.1. Solubility Test

Solubility of the extracted gum was evaluated qualitatively by stirring 10 mg of Okra powder in 10 mL water, acetone, chloroform, and ethanol (1% dispersion). Solubility was determined by visual observation of the solute.

#### 2.3.2. pH Determination

1% wt/vol dispersion of the sample in water was stirred consistently for 5 minutes and pH was determined using a pH meter.

#### 2.3.3. Moisture Content

Moisture content of Okra gum powder was conducted by measuring *≈*100 mg of powder using Mettler Toledo HR73 Halogen Moisture Analyzer, with loss on drying at 105°C.

#### 2.3.4. Viscosity

Viscosity of Okra gum at 1% and 0.5% concentrations was performed using the DV-III Ultra Programmable Rheometer with Brookfield Rheocalc Application Software.

#### 2.3.5. X-Ray Diffraction Analysis

X-Ray Diffraction was carried out on Inxitu Benchtop XRD/XRF Instrument at 250 exposures in ambient condition using Cu K*α* radiation.

#### 2.3.6. Thermal Analysis Thermogravimetric Analysis (TGA) and Differential Scanning Calorimetry (DSC)

Thermogravimetric measurements (TGA) of Okra powder were performed using TA instruments TGA 500 with heating scans of ambient temperature (21°C) to 900°C at an automated heating rate.

Perkin Elmer DSC6 was used to study the thermal characteristics of the gum. About 2.5 mg sample was placed in an aluminum pan and was scanned at −20°C to 230°C at a scanning rate of 10.00°C/min. Nitrogen was used as purged gas at a flow rate of 20 mL/min.

#### 2.3.7. Fourier Transform Infrared (FTIR)

The Fourier transform-infrared (FTIR) spectrum of the sample was recorded in FTIR Thermo Scientific in the range of 400–4000 cm^−1^, in attenuated reflection mode (ATR).

#### 2.3.8. Field Emission Scanning Electron Microscope (FESEM)

The morphology and nature of Okra gum were analyzed and observed using FESEM at 4000x and 5000x resolutions.

### 2.4. Formulation of Propranolol Sustained Release Tablet

One constant ratio of material for each formulation was used to prepare the tablets to compare the behavior of natural (Okra), semisynthetic (sodium alginate), and synthetic (HPMC) polymers in the development of sustained release dosage forms. 90 mg of material was used in each formulation, which represented 30% of the tablet weight, in accordance with the standard ratio of binder for sustained release systems (Table 1).

### 2.5. Granulation and Tablet Compression

Tablets were prepared using granulation method. First, propranolol hydrochloride salt, Okra gum/sodium alginate/HPMC, sodium bicarbonate, and lactose were weighed and mixed thoroughly with a spatula and sieved using the no. 30 mesh. Then, small drops of propanol were added to the mixture, uniformly mixed to form granules, and passed through sieve no. 25. The mass was then dried in a hot dry oven at 40°C for 40 minutes and again passed through sieve no. 25, whereby the mesh size will form suitable granule size to produce effective sustained release tablets. PEG and magnesium stearate were then added into the dried granules as a lubricant for the compression process. Granules were compressed with a compaction pressure of 2700 psi by using tablet-shaped punches on Enerpac Tablet Punch.

### 2.6. Evaluation of Propranolol Tablets

Based on previous studies, experiments were modified from the literature and conducted in accordance with British Pharmacopeia 2007.

#### 2.6.1. Thickness and Diameter

The thickness and diameter of tablets (*n* = 5) with each formulation were measured using Vernier Calipers. Average of tablets was determined and reported as mean ± standard deviation.

#### 2.6.2. Weight Variation

The average weight of 20 tablets was determined. Then, individual tablets were weighed and compared with the average. Results are reported as mean ± standard deviation.

#### 2.6.3. Hardness

The hardness of tablets (*n* = 5) was determined using a Monsanto hardness tester. The average hardness of tablets was reported as mean ± standard deviation.

#### 2.6.4. Friability

The friability of 12 tablets was determined using a Roche Friabilator (with a total tablet weight of 6.5 g). The friabilator was operated at 25 rpm per minute for 4 minutes (100 revolutions). The tablets were then weighed again (*W*_final_). The % friability was calculated as

(1) $$\begin{array}{c} F=\frac{\left(W_{\text{initial}}-W_{\text{final}}\right)}{W_{\text{initial}}}*100. \end{array}$$

#### 2.6.5. Swelling Index

This method was incorporated from Ravindran et al. [8] and slightly modified. Tablets were weighed individually and dispersed in 900 mL of pH 1.2 hydrochloric acid at 37.0 ± 0.5°C and 50 rpm rotation. At 30-minute, 1-hour, 2-hour, 3-hour, and 4-hour intervals, tablets were withdrawn and dabbed with filter paper to absorb excess buffer solution and then weighed again. Percentage swelling of tablets was expressed as the following:

(2) $$\begin{array}{c} \text{Swelling}\;\text{index}=\frac{W_{t}-W_{o}}{W_{o}}*\;100, \end{array}$$

where *W*_*o*_ represents the initial weight of tablet and *W*_*t*_ represents the weight of swollen tablet at time *t*.

#### 2.6.6. *In Vitro* Dissolution Studies

The release rate of tablets was determined using US Pharmacopeia 29 (USP29) Dissolution Testing Apparatus 2 (paddle method). The dissolution test was performed using 900 mL of 0.1 N hydrochloric acid (HCl), at 37 ± 0.5°C and 50 rpm. A sample (10 mL) of the solution was withdrawn from the dissolution apparatus at designated intervals, where withdrawn samples were replaced with fresh dissolution medium. Samples were filtered through a 0.45 *μ* membrane filter and the released drug absorbance was measured at 291 nm using a UV/visible spectrophotometer.

#### 2.6.7. Drug Release Kinetics

To investigate the drug release kinetics of all formulations, data obtained from *in vitro* release study was analyzed according to the zero order model, first order model, Higuchi's model, Hixson-Crowell model, and Korsmeyer-Peppas model (Table 4).

## 3. Results and Discussion

Extraction method using the described technique was employed since slicing Okra produced a higher amount of gum solute after 24 hours of homogenization. Acetone was used as drying agent as it is able to separate out the gum from its solute while preserving its main functionality as a hydrophilic binder. Characterization of the extracted Okra gum was performed to determine the physical and chemical attributes of the polymer.

### 3.1. Characterization of Okra Gum

Okra powder was shown to be sparingly soluble in water and insoluble in acetone, ethanol, and chloroform. An increase in solubility was observed when temperature was applied. However, Okra gum produced clumped gum in acetone and this indicated that acetone is a good precipitating and drying agent to produce dried Okra. Okra powder was observed to swell and form viscous dispersion when dispersed in water. The slightly soluble behaviour of Okra gum is useful in this formulation as the swellable and viscous dispersion represents a strong matrix polymeric system that is able to control the release of highly soluble propranolol hydrochloride drug in the stomach.

The pH of Okra gum is 6.59. Okra gum is known to have maximum viscosity at a neutral pH range, which helps in the retarding effect for the development of sustained release tablets. Neutral pH also causes minimum irritation to the gastrointestinal tract and is suitable for uncoated tablets [4]. Moreover, the neutral pH of Okra gum will not alter the pH of Okra tablet that is formulated with propranolol hydrochloride, which is a weak basic drug.

Moisture content of Okra gum is 14.83%, indicating that Okra gum contains bound moisture to the polymer. This is due to the polymer adsorption sites that is able to bind water molecules to the polysaccharide structure via hydrogen bond [9], which leads to a larger permeability of hydrophilic materials [10]. When Okra particles are brought into close proximity, the water sorption will interact, resulting in the formation of a strong interparticular attraction between the particles. Bound moisture will affect the compressibility of tablets by formation of moisture film on the particles upon applied pressure from the tablet compression machine. This layer of moisture may also lubricate the powder and allow easy flow of tablets by reducing friction on the die wall during tablet ejection [2]. The film of moisture on the Okra powder also allows the powder to stick to each other better and produce more intact tablets.

**Figure 3:**
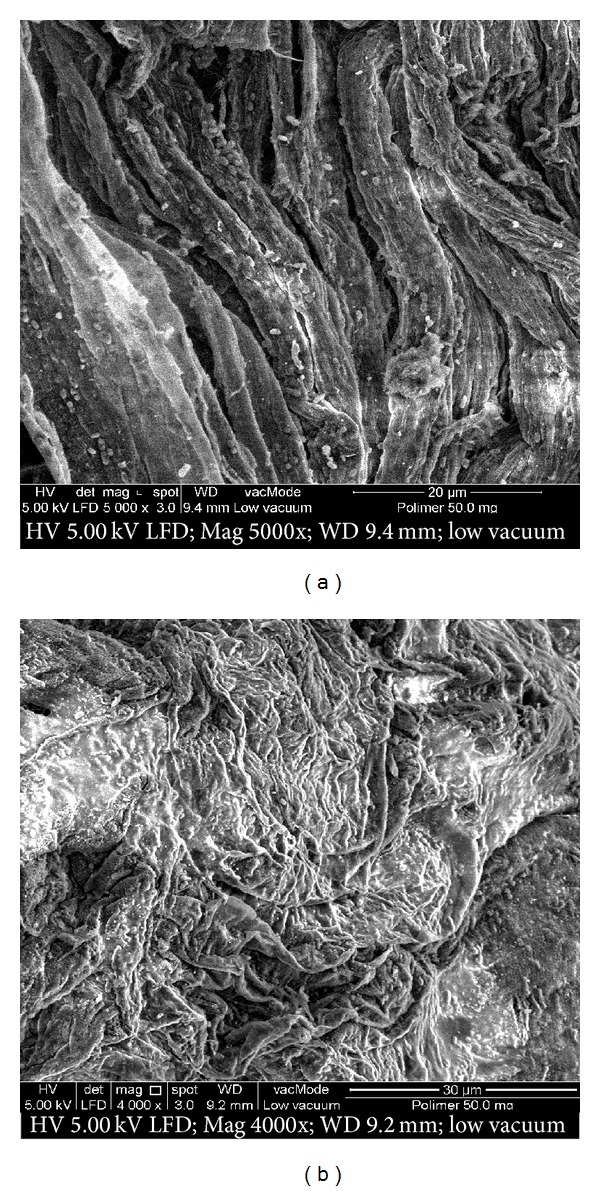
Surface morphology of Okra using field emission scanning electro microscope (FESEM).

Viscosity of Okra gum 1% solution is higher (228.78 cP) compared to the viscosity of Okra gum at a lower concentration (0.5% solution) which is 62.32 cP. This indicates that Okra gum has higher viscosity at a higher concentration. The higher the viscosity of the gum, the more sticky it is and this produces tablets with slower drug release and better tensile strength [11]. The gum with a higher degree of stickiness creates a more dense material with heavier cross linkage of molecules; therefore it is able to hold the ingredients in a tablet more efficiently and produce tablets with better retarding effects. This can be seen in the visual image of Okra gum from FESEM analysis (Figure 3), where the structure of Okra appears to be compact, thus preparing minimal matrix space, enabling the ingredients to be contained in the tablet more efficiently.

**Figure 4:**
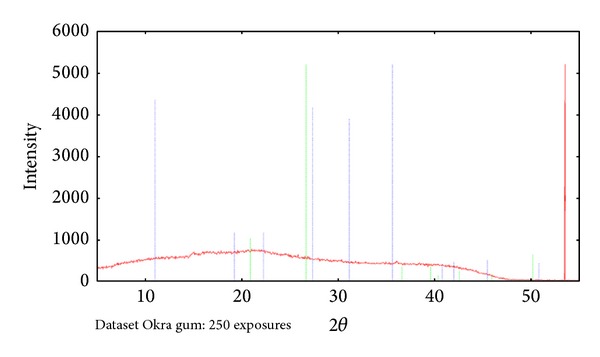
X-ray diffraction analysis of Okra.

**Figure 5:**
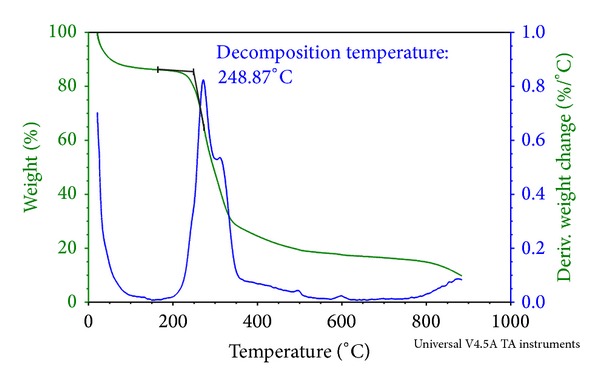
Thermogravimetric analysis (TGA).

XRD analysis of Okra as can be seen in Figure 4 showed that it consists of amorphous and crystalline structure. The broad distribution that could be seen from the X-ray diffraction spectrum indicates the amorphous nature of the polymer. This is due to the scatterings of X-rays by atoms that are randomly distributed in a wide range, visualizing a broad bump. The polymer pattern that is presented by the XRD spectrum shows the transformation from amorphous to crystalline structure, which can be seen from the broad halo to the distinct crystalline peak. Crystalline structures are formed by aligned atoms that are positioned in periodic arrangements resulting in high intensity peaks upon the hitting of X-ray beams onto the lattice planes.

**Figure 6:**
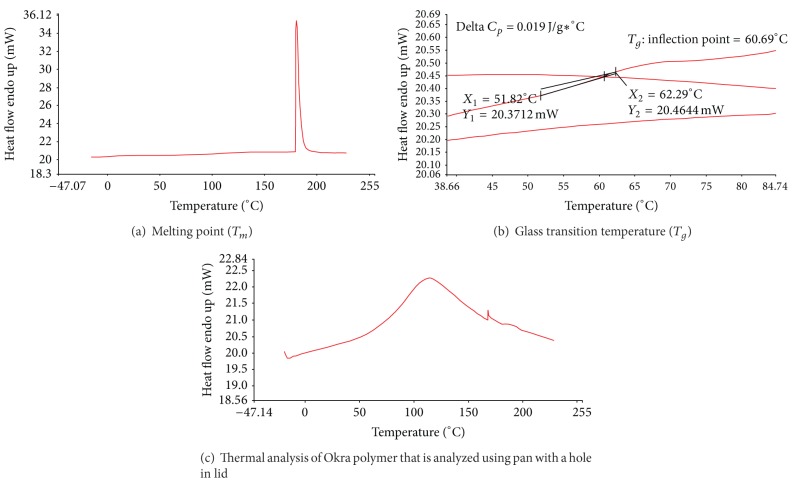
Differential scanning calorimetry (DSC).

For the thermal characteristics of Okra as demonstrated in Figures 6(a) and 6(b), the glass transition temperature (*T*_*g*_) and melting point (*T*_*m*_) of Okra are 60°C and 180°C, respectively, based on the analysis conducted using DSC. As the characteristic of Okra is learned to be a mixture of amorphous and crystalline structures from the XRD spectrum, glass transition that is ordinarily present in amorphous and semicrystalline structures was detected. Below glass temperature, the structures of molecules are in a glassy state where they are frozen in place or slightly vibrating. At the glass transition temperature, the molecules start to move but are subjected to only vibration, whereas, above the glass temperature, molecules are converted from glassy to rubbery state where they experience higher mobility [12]. Since *T*_*g*_ of Okra powder is 60°C, it therefore exists in a glassy state at room temperature and considered to be stable during the experiment process and storage as it does not experience any extrusive chemical movements. Okra is preferably stored at or below room temperature in a dry environment to minimize occurrence of any chemical changes to the structure of molecules, thus preserving its quality and functionality [13]. Okra gum contains bound moisture, as detected from the moisture content analysis; therefore, in order to measure the moisture activity of the molecules, thermal analysis with DSC was conducted using a pan with a hole in its lid. A broad peak was distinguished at 120°C as can be seen in Figure 6(c). This broad peak represents the evaporation activity of bound moisture in the Okra gum during the DSC run and it was released through the hole of the aluminium lid. The evaporation temperature is slightly higher than boiling temperature as more amount of heat is needed to break up the ionic bond between water molecules and the polysaccharide linkages. This concurred with the work done by Mukherjee and Rosolen [14] on thermal analysis of gelatin.

The main components of Okra which are galactose, rhamnose, and galacturonic acid were determined in the spectrum of FTIR analysis as shown in Figure 6. A broad peak at 3335.44 cm^−1^ was found in the spectrum, indicating the presence of aromatic sugar groups with O–H as the main functional group, which was found in the 3 main components of Okra. O–H groups are able to bind with water molecules and produce bound moisture to the polymer components. The existence of O–H groups represents the hydrophilic characteristic that is present in the polysaccharide. The medium peak that is visible at 2938.69 cm^−1^ represents C–H stretch that exist in galactose and rhamnose. The small peak at 1719.07 cm^−1^ shows the presence of C=O stretch that can be found in the constituent of galacturonic acid while the identical small peak at 1418.06 cm^−1^ indicates C–H bend which is a constituent of galactose and rhamnose. The frequency of 1200–1000 cm^−1^ indicates C–O stretch bonds which are present in the aromatic compounds of galactose, rhamnose, and galacturonic acid. The methyl, carbonyl, and hydroxyl functional groups that are present in the chemical structure of Okra are constituents of carbohydrate molecule, which is concluded to be the main backbone of the polymer (Table 2).

### 3.2. Evaluation of Propranolol Tablets

The physical characteristics of 5 tablets are represented as mean ± standard deviation. Based on Table 3, the size of tablets was observed to be uniform as shown by the low standard deviation value for thickness, diameter, and weight variation. For the tensile strength evaluation of the tablets, Okra gum exhibited the highest hardness and lowest friability compared to HPMC and sodium alginate. This indicates that Okra gum produces stronger tablets and is more capable of protecting tablets against capping and lamination [3]. Due to its hygroscopic characteristics, Okra gum acts as a good binder as it is able to retain moisture that helps in reducing stickiness to the tablet punches during compression process [2]. It is also a good plasticizer as it forms a smooth film that acts as a coating material for the tablets.

**Figure 7:**
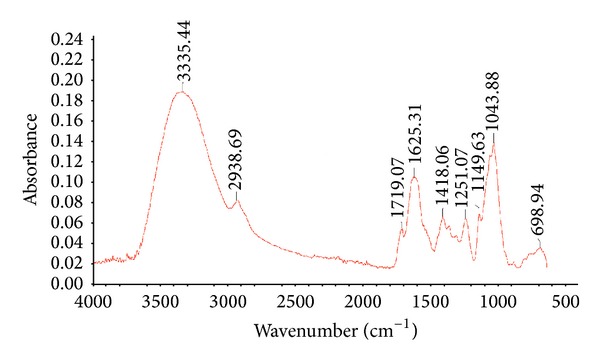
Composition of Okra polymer by FTIR (Fourier transform infrared spectroscopy).

In observation of tablet's swelling index and drug release as referred to Figure 7, the weight of tablets that were formulated with Okra was seen to increase up to 3 hours and decreased at the 4th hour. This shows that Okra gum was able to absorb water and swelled until the 3rd hour and erosion of swelled gel layer began to occur at the 4th hour. The swelling of tablet occurred from the formation of matrix layer by the polymer around the tablet, enabling it to control drug release. This matrix layer begins to erode when disintegration occurs faster at the 4th hour. Although the disintegration of tablets occurred more at the 4th hour, dissolution profile showed that the drug was released in a moderate and consistent manner up to 24 hours. This occurs due to the high viscosity of Okra that is able to act as a compatible retardant to control the drug release in a steady pattern for a prolonged period of time [11]. For the swelling index of tablets that were formulated with HPMC and sodium alginate, the weight of tablets was seen to decrease at the first hour. This indicates that HPMC and sodium alginate were not that successful in allowing the tablets to swell for the purpose of controlling the rate of release. This could also be seen from the rapid drug release of tablets formulated with these 2 polymers during the first 3.5 hours of dissolution studies (Figure 9).

Dissolution studies were carried out in HCl only, as propranolol hydrochloride is documented to be a weak basic drug (p*K*_*a*_ = 9.5), where it is freely soluble and ionized in acidic environment. In previous studies, it was learned to be highly soluble in pH 1.2 (225 mg/mL) and less soluble in pH 6.8 (130 mg/mL) [15]. The high solubility causes rapid release and might lead to inflammation and ulceration of the stomach lining. Because of these characteristics, Okra polymer is being used to sustain the release of propranolol hydrochloride in the aqueous acidic environment of *in vitro* study, which represents the upper part of the gastrointestinal tract in order to minimize side effects and enhance its therapeutic value by releasing medication moderately and consistently throughout the 24-hour release.

The rate of drug release was observed to be the fastest in tablets formulated with sodium alginate where it reached 70% of release in the first 15 minutes and released moderately until it reached maximum release at 3.5 hours. For HPMC tablets, drug release was seen to be rapid for the first 1.5 hours and they experienced moderate release up to its maximum release at 3.5 hours. As for Okra tablets, the release was observed to be relatively consistent until it reached maximum release up to 24 hours. Chodavarapu et al. have applied Okra in Metformin hydrochloride floating tablets with an approximately similar ratio and have exhibited faster rate of release where it reached maximum release at 8 hours [16] whereas Kalu et al. have utilized Okra in controlled release Paracetamol tablets for up to 6 hours [11]. This difference is believed to be due to the diversity of Okra sources, differences in extraction technique, and the variety of tablet formulation and granulation techniques, as well as compression method.

**Figure 8:**
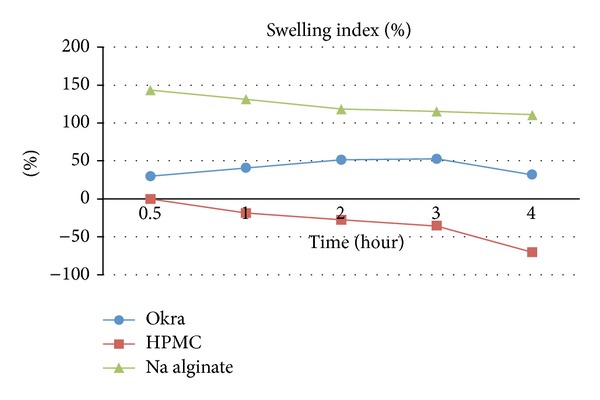
Swelling index of tablets.

**Figure 9:**
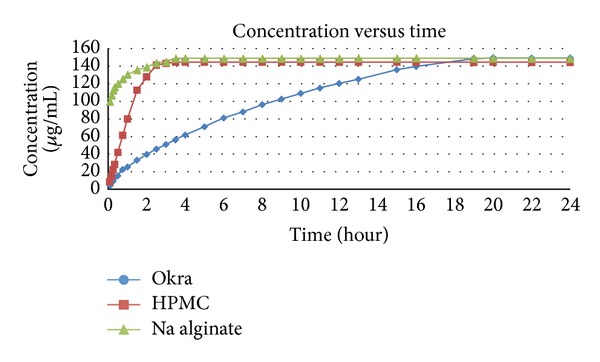
Drug release of tablets.

As for the analysis of drug release according to various kinetic models, the regression values derived from the formulas of respective kinetic models were determined, whereby the highest regression (*r*) value indicates its release pattern. The *r* value for tablets formulated with Okra and sodium alginate mechanism of drug release was found to be in accordance with the Korsmeyer-Peppas model. This model is used to express water soluble drug that undergoes swelling and diffusion from controlled release tablets with polymeric structure [17]. For Okra tablets, the drug release mechanism conformed to the non-Fickian transport, which indicates the occurrence of swelling and diffusion of tablets. For tablets with sodium alginate, the drug release complied with supercase transport-2, determining that tablets underwent the process of swelling throughout its release. Tablets containing HPMC were concluded to follow the first order model, where its dosage form can be described as containing water soluble drug in porous matrix [17] and having slow release, controlled by matrix erosion (Figure 8).

## 4. Conclusion

It can therefore be concluded that Okra gum is a natural semicrystalline polysaccharide, which is effective as a retarding polymer to develop sustained release tablets. It is able to formulate propranolol hydrochloride tablets up to 24 hours of release as compared to HPMC and sodium alginate as retarding agent.

## Figures and Tables

**Table 1:** Prepared formulation of propranolol sustained release tablets.

Propranolol	150	150	150
Okra	90	—	—
HPMC	—	90	—
Sodium alginate	—	—	90
Lactose	252	252	252
Polyethylene glycol	5	5	5
Magnesium stearate	3	3	3

Total	500	500	500

F1: Okra gum as binder.

F2: HPMC—hydroxylmethylcellulose as binder.

F3: Sodium alginate as binder.

**Table 2:** Characterization of Okra gum.

Solubility test	Slightly soluble in water, insoluble in acetone, ethanol, and chloroform
pH	6.59
Moisture content	14.83%
Viscosity	0.5% concentration: 62.32 cP 1% concentration: 228.78 cP
Thermal analysis	*T*_*g*_: 60°C *T*_*m*_: 180°C

**Table 3:** Physical characteristics of tablets.

Parameter	Okra	HPMC	Na alginate
Thickness (mm)	5 ± 0	5.3 ± 0	5 ± 0
Diameter (mm)	9.65 ± 0	9.65 ± 0	9.65 ± 0
Weight variation (mg)	495.73 ± 1.77	491.2 ± 0.51	497.53 ± 2.85
Hardness (N)	283.33 ± 2.49	85.33 ± 2.05	28.33 ± 3.09
Friability (%)	0.01	0.57	9.47

**Table 4:** Regression analysis values (*r* value) for *in vitro* drug release data following different kinetic models.

Kinetic model		Okra	HPMC	Na alginate
Zero order	*r* value	0.9858	0.9433	0.9336
First order		0.9879	0.9911	0.9698
Higuchi model		0.9989	0.9813	0.9826
Hixson-Crowell model		0.9019	0.8998	0.9202
Korsmeyer-Peppas model	*r* value	0.9990	0.9896	0.9994
	*n* value	0.6385 Non-Fickian transport		0.1059 Supercase 2 transport
